# Effects of an Online Mind–Body Training Program on the Default Mode Network: An EEG Functional Connectivity Study

**DOI:** 10.1038/s41598-018-34947-x

**Published:** 2018-11-16

**Authors:** Dasom Lee, Do-Hyung Kang, Na-hyun Ha, Chang-young Oh, Ulsoon Lee, Seung Wan Kang

**Affiliations:** 1 Department of Psychiatry, Seoul National University College of Medicine, Seoul, Republic of Korea; 2 Department of Psychiatry, Seoul National University Hospital, Seoul, Republic of Korea; 3 Department of Brain-based Emotion Coaching, Global Cyber University, Seoul, Republic of Korea; 4 Data Center for Korean EEG, College of Nursing, Seoul National University, Seoul, Republic of Korea; 5 iMediSync Inc., Seoul, Republic of Korea; 6 Emotional Information andCommunication Technology Industrial Association, Seoul, Republic of Korea

## Abstract

Online mind–body training (MBT) programs can improve the psychological capabilities of practitioners. Although there has been a lot of effort to understand the neural mechanisms underlying the therapeutic effects of meditation, little is known about changes in electroencephalographic (EEG) functional connectivity that accompany mind-body training. The present study aimed to investigate how an online MBT program alters EEG functional connectivity in the default mode network (DMN). We assessed a group of healthcare providers, including 14 females who participated in the 4-week MBT program and 15 females who underwent a 4-week of waiting period. EEG data and information about psychological states were obtained at baseline and 4 weeks. The result was that the intervention group showed significant reductions in anxiety and trait anger that were accompanied by increased global DMN network strengths in the theta and alpha (but not beta and delta) frequency bands; these changes were not observed in the control group. Other variables including state anger, positive and negative affect, and self-esteem have not been changed over time in both groups. These findings suggest that practicing the mind-body training could have a relevance to the functional differences in network related to stress and anxiety reaction.

## Introduction

Mindfulness, which is characterised as focusing on the present moment without judgment or mind wandering, has been intensively studied, and its beneficial impacts on various psychological states, including anxiety, the stress response, anger expression, and mood disorders, have been demonstrated^1–6^. Mind–Body Training (MBT), the combination of mindfulness and movement-based meditation, was designed to concentrate on bodily sensations, facilitate relaxation, and release negative emotions in the body through natural rhythmic movements^7^. Accumulating evidence suggests that this MBT program is an effective psychological intervention for the relief of stress responses^8–11^. In addition, it has been reported that the effects of the *online* MBT programs were comparable to those of the offline MBT^8,12,13^. For example, after 8 weeks of online participation in this MBT program, participants exhibited improvements in stress, coping strategies, anger, emotional intelligence, negative affect, and resilience^8^.

Studies investigating the neurophysiological mechanisms underlying the therapeutic effects of various meditation techniques have indicated that powers in the theta and alpha bands increase following meditation^14^. For example, increases in alpha power are observed during meditation^15–18^ as well as during a resting state^19–22^. However, few studies have investigated changes in global functional connectivity (FC) for each of these frequency bands. Recently, advances in brain network analysis have been employed to explore network changes in resting-state FC using various modalities to investigate intrinsic brain activity. Bassett and Bullmore (2006)^23^ reported that brain networks operate dynamically in a critical state and facilitate the rapid adaptive reconfiguration of neuronal assemblies in support of changing cognitive states. A reorganisation of the resting-state FC network has even been observed during simple finger movements, which suggests the utility of the resting-state FC network as a functional measure of the brain network reflecting intrinsic brain activities. So far, functional magnetic resonance imaging (fMRI) has been commonly used to investigate the FC at resting state. Recent neuroimaging studies of meditation have shown that meditation practices accompany the changes in the brain area such as the prefrontal cortex (PFC), anterior cingulate cortex (ACC), striatum, amygdala, and insula^7,24–28^. However, few studies have investigated the impact of meditation practice on the EEG FC at different time series and frequency bands^29–31^. Emerging evidence has shown that EEG is a useful tool to explore functional connectivity at resting state^32–34^. With EEG analysis, it is possible to evaluate changes in global FC at resting state in several frequencies (delta, theta, alpha, beta 1, beta 2), and thus, investigate the effects of MBT on dynamic brain networks.

The present study aimed to investigate the effects of a MBT program on the default-mode network (DMN), which is a representative resting-state FC network, to further elucidate how, from a resting-state FC network perspective, MBT improves markers of mental health. To this end, the present study tracked the changes in EEG FC signals and the responses to self-administered psychological questionnaires in a group of females who participated in the 4-week MBT intervention group and a group of females who underwent a 4-week waiting period.

## Results

**Figure 1:**
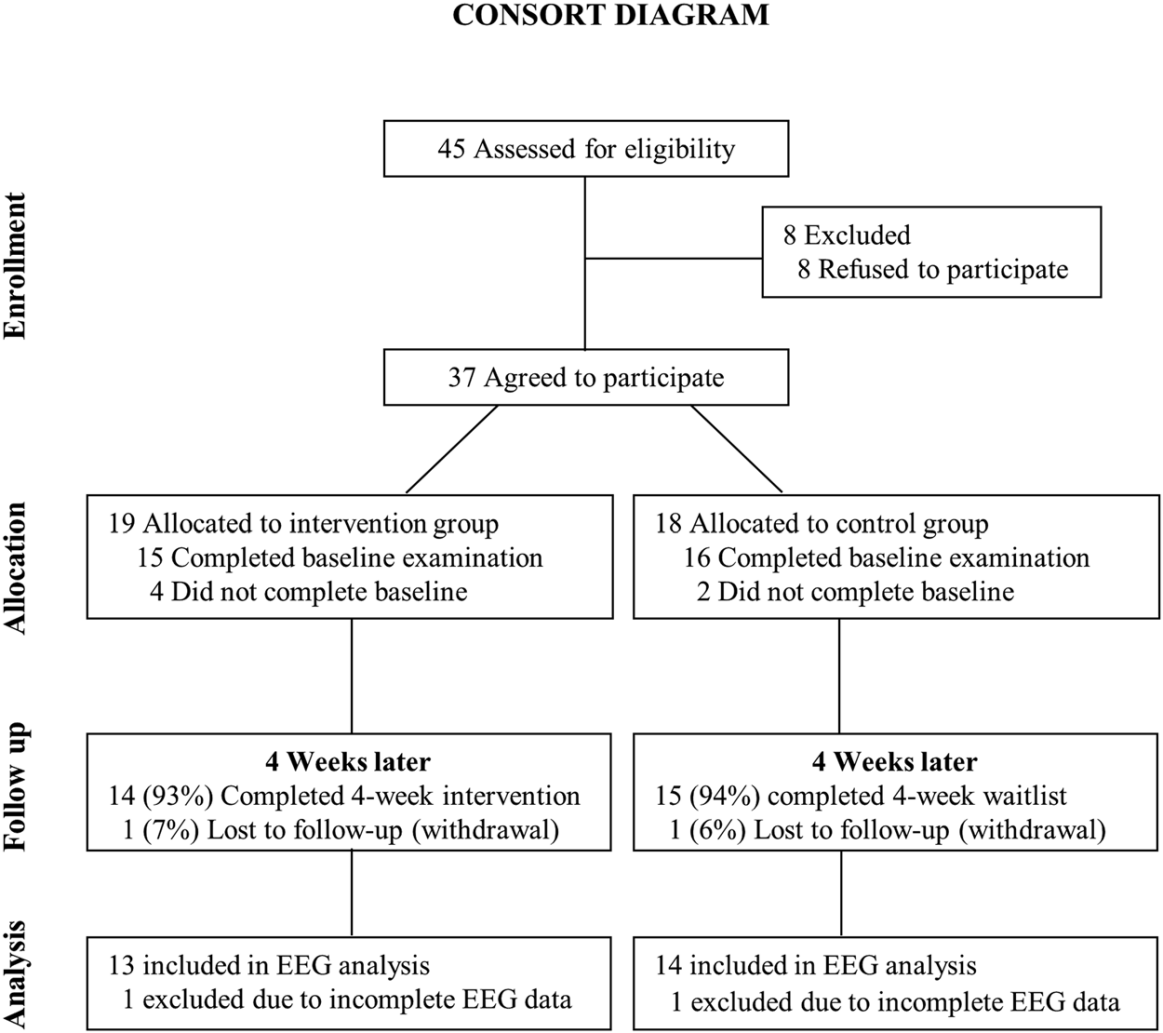
Flow of the study.

As shown in Fig. 1, 37 people participated in the experiment. They were randomly assigned to the MBT group (N = 19) and the control group (N = 18). Four participants of the MBT group and two participants of the control group did not complete baseline examination. After 4 weeks of time, 1 participants of the MBT group and 1 participants of the control group were lost to follow up. Thus, 29 participants completed the 4 weeks follow-up assessment, 14 in the MBT group and 15 in the control group (Fig. 1).

Excluding two participants due to incomplete or missing questionnaire data (1 in the MBT group and 1 in the control group), per-protocol analyses were conducted including 13 MBT participants and 14 healthy controls. In addition, the FC network analyses included 13 MBT participants and 14 healthy controls, ruling out two participants with incomplete EEG data (1 in the MBT group and 1 in the control group),

### Psychological Measures

At baseline, there were no significant differences between the groups in age, psychological symptoms (SCL-90), self-esteem (RSES), anger expression (STAXI), or positive and negative affect (PANAS) (*p*s > 0.05) (Table 1). In addition, the two groups did not differ in degree of occupational stress at baseline (KOSS; *t* = −0.60, *p* = 0.56), and there was no significant change in occupational stress over time for both groups (MBT: *t* = 1.90, *p* = 0.08; control group: *t* = −1.38, *p* = 0.19).

**Table 1:** Descriptive Statistics for outcome measures.

	MBT (N = 13)		HC (N = 14)		RM ANOVA		
	PRE	POST	PRE	POST	Group (*p*)	Time (*p*)	Group x Time (p)
Age, years	36.77 (8.18)		34.07 (6.18)				
SCL-R-90							
*Somatization*	0.72 (0.54)	0.50 (0.41)	0.71 (0.44)	0.69 (0.53)	0.601	0.113	0.170
*Obsessive-compulsive*	0.83 (0.67)	0.64 (0.40)	1.24 (0.67)	1.21 (0.74)	**0.042**	0.235	0.361
*Interpersonal sensitivity*	0.83 (0.69)	0.67 (0.47)	1.18 (0.66)	1.25 (0.86)	0.07	0.634	0.226
*Depression*	0.85 (0.86)	0.56 (0.47)	1.27 (0.87)	1.29 (1.02)	0.067	0.231	0.168
*Anxiety*	0.72 (0.72)	0.40 (0.48)	0.7 (0.8)	0.84 (0.87)	0.130	0.463	**0.001**
*Hostility*	0.76 (0.64)	0.45 (0.52)	0.81 (0.85)	0.81 (0.92)	0.449	0.119	0.117
*Phobic anxiety*	0.22 (0.37)	0.14 (0.21)	0.30 (0.40)	0.38 (0.74)	0.368	0.971	0.280
*Paranoid ideation*	0.49 (0.59)	0.49 (0.63)	0.70 (0.54)	0.76 (0.76)	0.283	0.769	0.769
*Psychoticism*	0.26 (0.51)	0.17 (0.30)	0.44 (0.36)	0.46 (0.53)	0.162	0.505	0.212
RSES	30.92 (6.53)	31.31 (5.98)	29.57 (4.70)	30.57 (4.26)	0.608	0.214	0.576
PANAS							
*Positive affect*	26.69 (8.64)	29.69 (8.73)	26.57 (7.14)	25.57 (5.76)	0.447	0.346	0.066
*Negative affect*	25.92 (6.40)	24.15 (7.36)	25.71 (8.62)	26.86 (7.61)	0.646	0.785	0.211
STAXI							
*State anger*	12.00 (3.16)	13.69 (8.16)	10.71 (1.14)	12.50 (4.65)	0.372	0.201	0.972
*Trait anger*	23.08 (4.89)	20.38 (5.49)	21.57 (6.82)	22.86 (5.91)	0.823	0.317	**0.008**

Data are given as the mean (standard deviation). SCL-R-90, Symptom Checklist-90-Revised; RSES, Rosenberg Self-Esteem Scale; PANAS, Positive and Negative Affect Schedule; STAXI, State-Trait Anger Expression Inventory.

To examine the effects of the MBT program, a rm ANOVA assessing the psychological outcomes was conducted with group as a between-subjects variable and time as a within-subject variable. Analyses of the SCL-R-90 subscales revealed a significant time × group interaction for anxiety (F_[1, 25]_ = 14.69, *p* = 0.001), and post hoc analyses revealed that anxiety was alleviated in the MBT group (*t* = 3.51, *p* = 0.004) but not in the control group (*t* = −1.75, *p* = 0.103); there were no other significant group differences across time. There was no significant main effect of group or age for the SCL-R-90 subscales (*p*s > 0.05), except that the main effect of group was significant for obsessive-compulsive (*p* = 0.042). The same analyses were conducted to investigate state and trait anger. There was a significant group difference across time for trait anger (F_[1,25]_ = 8.343, *p* = 0.008) but not for state anger (F_[1,25]_ = 0.001, *p* = 0.972). Post hoc analyses showed that trait anger was significantly reduced in the MBT group (*t* = 3.35, *p* = 0.006) but not in the control group (*t* = −1.172, *p* = 0.262).

No interaction effects were observed for self-esteem (RSES: F_[1,25]_ = 0.321, *p* = 0.576) or positive/negative affect (positive: F_[1,25]_ = 3.694, *p* = 0.066; negative: F_[1,25]_ = 1.646, *p* = 0.211), which indicates there were no significant differences between the two groups in terms of self-esteem and positive/negative affect. Taken together, the present findings demonstrate the beneficial effects of the MBT program on anxiety and trait anger.

### EEG

There was no baseline difference between two groups in age (MBT: mean 33.07 ± standard deviation 5.51 years; Controls: 37.85 ± 7.91 years), alpha (MBT: 18.31 ± 3.95; Controls: 20.33 ± 2.97), beta 1 (MBT: 20.45 ± 3.64; Controls: 19.74 ± 2.08), beta 2 (MBT: 19.65 ± 4.70; Controls: 20.17 ± 2.99), theta (MBT: 18.74 ± 3.53; Controls: 21.27 ± 3.04), and delta (MBT: 19.54 ± 2.92; Controls: 19.44 ± 2.57) frequency band.

**Figure 2:**
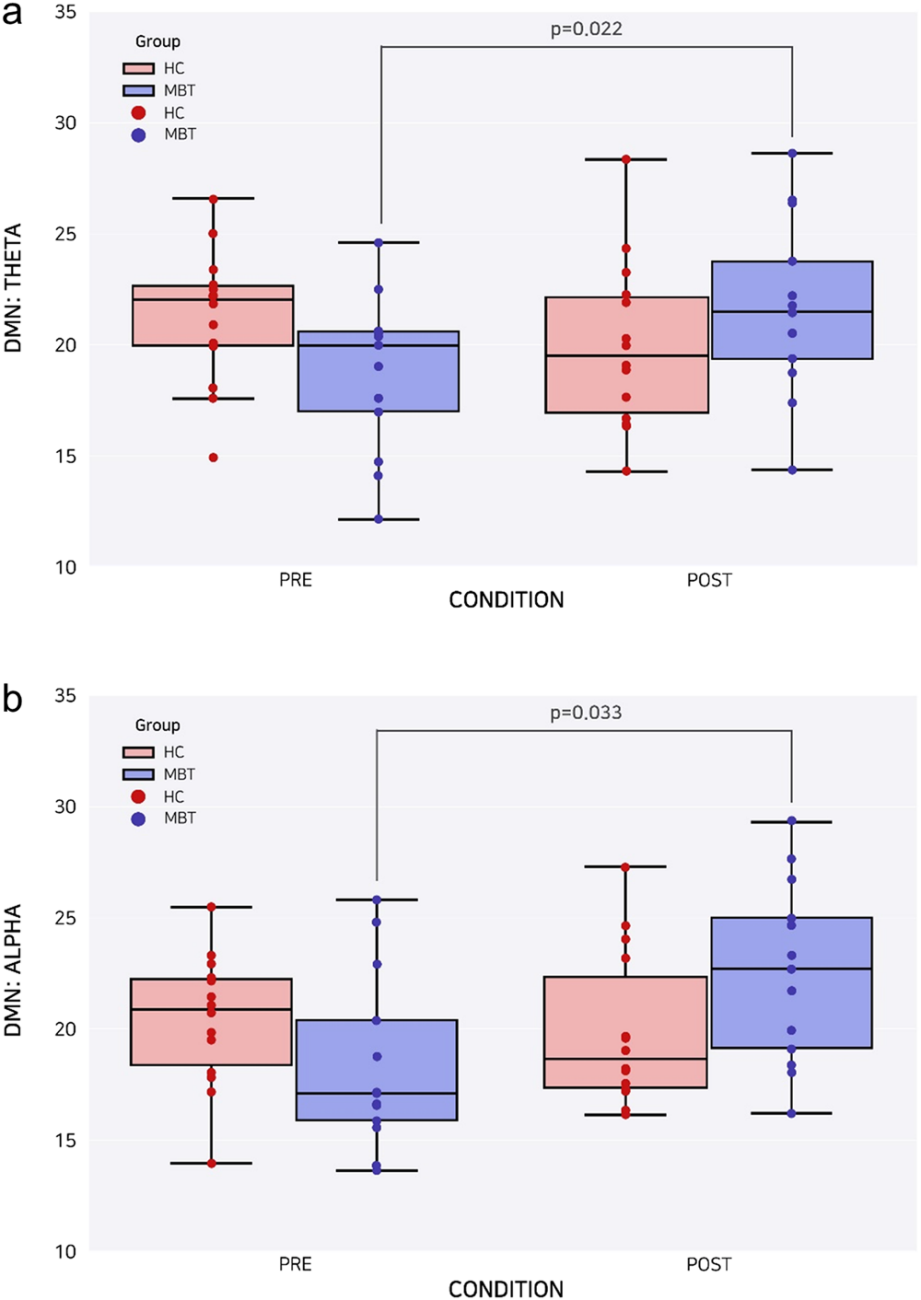
Changes in the global FC values of DMN for the HC and MBT groups in the PRE and POST states at the theta (**a**) and alpha (**b**) frequency bands. DMN = Default Mode Network; HC = Healthy Control; MBT = the MBT (mind-body training) group; PRE indicates the first EEG recordings and POST indicates the second EEG recordings 4-weeks after the first EEG recordings.

**Figure 3:**
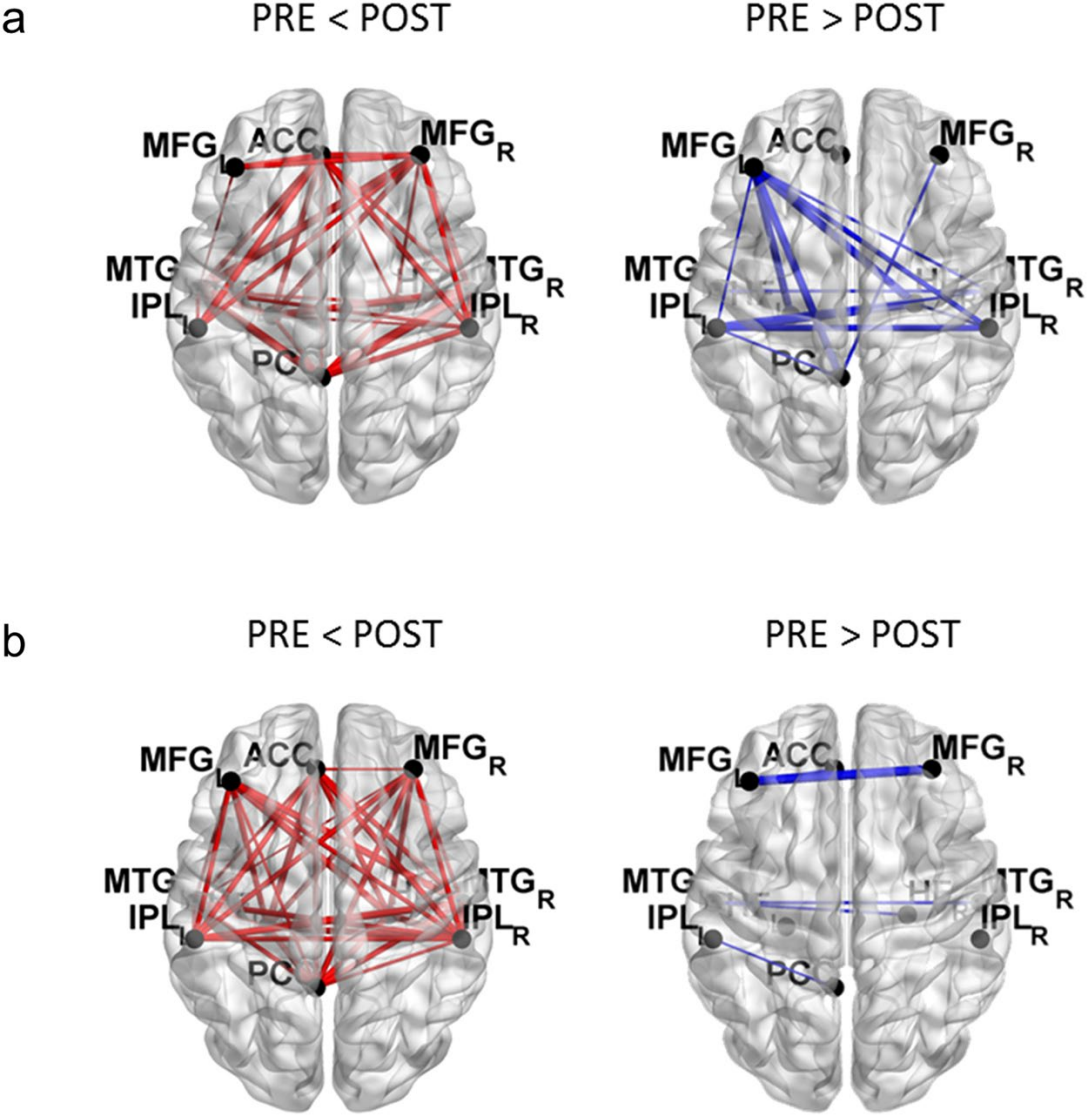
Network changes of the group averaged FC values at each pairs of connections within the DMN of the MBT group in the theta (**a**) and alpha (**b**) frequency bands. Red line indicates stronger connectivity in the POST than in the PRE, and blue line indicates weaker connectivity in the POST than in the PRE. MFG = Middle Frontal Gyrus; ACC = Anterior Cingulate Cortex; MTG = Middle Temporal Gyrus; IPL = Inferior Parietal Lobule; PCC = Posterior Cingulate Cortex.

A 2 × 2 rm ANOVA showed significant time × group interactions in the theta (*p* = 0.015, Table 2 and Fig. 2a) and alpha (*p* = 0.037, Table 2 and Fig. 2b) frequency bands, which reflect significantly different changes between the two groups over time. Post hoc analyses revealed increased theta global FC (*p* = 0.022) after 4 weeks of MBT, but no significant difference in global FC values over time was observed in the HC group. Similarly, the MBT group exhibited increased alpha global FC (*p* = 0.033), whereas the HC group did not differ in terms of EEG data over time. The descriptive network changes of the group-averaged FC values within the DMN of the MBT group in the theta (Fig. 3a) and alpha (Fig. 3b) frequency bands are provided.

**Table 2:** Global FC values of the HC and MBT groups in both PRE and POST states in the theta and alpha frequency bands.

	Global FC of DMN (mean ± S.D.)			
	PRE		POST	
	HC	MBT	HC	MBT
Theta	21.27 ± 2.04	18.74 ± 3.53	19.97 ± 3.78	21.66 ± 3.95*
Alpha	20.33 ± 2.97	18.31 ± 3.95	19.80 ± 3.50	22.44 ± 4.06*

Note: *p < 0.05 in the comparison of global FC of DMN between PRE and POST; DMN = Default Mode Network; FC = Functional Connectivity; HC = Healthy Control; MBT = Mind-Body Training; S.D. = standard deviation; PRE indicates the first EEG recordings and POST indicates the second EEG recordings 4-weeks after the first EEG recordings.

## Discussion

The present study demonstrated the beneficial effects of a 4-week online MBT program on FC in the DMN as well as on psychological state. Participants who completed the 4-week online MBT program showed significant reductions in anxiety and trait anger, while there was no change in state anger, positive and negative affect, self-esteem, and other psychiatric symptoms. Furthermore, the strength of the global DMN network in the MBT group showed increases, especially in the theta and alpha (but not in the beta and delta) frequency bands, after online MBT. In contrast, the controls did not show any improvement in psychological state or brain FC.

The influence of the MBT program on brain FC observed in the present study is in line with the findings of previous studies, which found that meditation primarily influences the theta and alpha bands^14^. Increases in the alpha and theta bands following meditation can be interpreted in terms of anxiety levels because these bands are related to stress and the anxiety response. For instance, alpha activity is negatively correlated with anxiety levels and positively correlated with calmness and positive affect^15,35,36^. However, it should be noted that higher levels of alpha activity during a resting state in meditators compared to controls may reflect selection bias^14^; that is, activity in the alpha band can be attributed to personal traits (e.g., likelihood of choosing to meditate) rather than to meditation itself. On the other hand, the present study recruited meditation-naïve participants for each group, and only the intervention group participated in the online MBT program. After 4 weeks, changes in FC were observed in the intervention group but not the control group. Thus, the present results provide evidence of a more direct causal relationship between mind-body training and neurophysiological changes. Taken together, our study adds to previous findings by confirming that mind-body training enhances global DMN network strengths in the theta and alpha frequency bands.

A notable clinical implication of the present study is its confirmation of the effectiveness of the *online* MBT program. That is, participation in the MBT program for just 4 weeks via the Internet or a smartphone (i.e., without contacting the trainer) was sufficient to strengthen FC in the brain. Furthermore, the participants in the present study were employed as healthcare service representatives, and thus, more likely to suffer from high levels of occupational stress and emotional burnout^37^. Because the online program used in the present study was effective in reducing anxiety and trait anger, it is expected to contribute to efforts to relieve stress in workplace.

The present study has a methodological limitation that should be addressed. Although strengthened FC was observed in the theta and alpha frequency bands following MBT, it was beyond the scope of the study to investigate the degree of the observed differences in the DMN. Future studies should investigate how each type of brain connection is affected by meditation. In addition, the current study has limitations in the way that the sample size was very small and active control group was lacking, which can limit the generalisability of our findings. Furthermore, since the effects of MBT can be largely influenced by the type of meditation, the level of experience, and subjects characteristics (e.g., female or male, healthy or mentally-ill), further investigation is required to make more definitive conclusions on the effect of MBT program in neurophysiological perspective.

In conclusion, the present study demonstrates that this short-term online MBT program has induced neurophysiological and psychological changes in the body. These findings can add to accumulating evidence for a possible causal relationship between MBT and FC in the brain. Taken together, these results provide additional proof that meditation-based MBT positively influences practitioners in terms of neurophysiology as well as psychology.

## Methods

### Subjects

We collected healthcare providers for 4-week mind-body training program. All participants were female and recruited from a hospital through announcements posted on several bulletin boards. As a healthcare provider, most of their work consisted of caring or helping patients and other workers in the hospital. Exclusion criteria included a history of psychosis, head trauma or neurological disorders. Subjects who were currently participating in meditation in the previous 3 months were excluded. Written informed consent was obtained from all participants, and the study protocol was approved by the Seoul National University Hospital Institutional Review Board (H-1611-037-806). All procedures were performed in accordance with the relevant guidelines and regulations.

### MBT Program

The intervention group (MBT) participated in an online MBT program via the Internet or their smartphone at either their home or their workplace once per day, 5 days per week, with each daily session lasting 8 minutes. After watching an online video, participants were instructed to follow the MBT expert and to record their daily practice on the paper provided. The program consisted of movement-based meditation, which was designed to calm one’s mind, let negative emotions out, and to concentrate on bodily sensations^7–9^. The protocol of the program is described in detail in Supplementary Note.

### Psychological Assessments

The Symptom Checklist-90-R (SCL-90-R) was used to assess psychological distress and symptoms of psychopathology. This measure consists of 90 items that evaluate somatization, obsessive–compulsive behavior, interpersonal sensitivity, depression, anxiety, hostility, phobic anxiety, paranoid ideation, and psychoticism. This questionnaire has been widely used to measure the outcomes of psychiatric and psychological interventions^38^.

The Korean Occupational Stress Scale (KOSS) is used to measure perceived stress related to one’s work environment^39^. This scale consists of 43 items that evaluate physical environment, job demands, insufficient job control, job insecurity, interpersonal conflict, organisational system, lack of reward, and occupational climate. It is scored using a conventional Likert scale (1–4) with higher scores indicating higher levels of occupational stress. Additionally, the Rosenberg Self-Esteem Scale (RSES), which is a 10-item self-report scale, was administered to measure self-esteem^40^, and the State–Trait Anger Expression Inventory (STAXI) and Positive and Negative Affect Schedule (PANAS) were used to evaluate anger expression and affect, respectively^41,42^.

### EEG

All EEG signals were recorded with a Mitsar-EEG 202 (Mitsar Ltd.; St. Petersburg, Russia) using 19 surface electrodes (Fp1, Fp2, F7, F3, Fz, F4, F8, T3, C3, Cz, C4, T4, T5, P3, Pz, P4, T6, O1, and O2) mounted on a cap (Electro-cap International Inc.; Eaton, Ohio, USA) according to the International 10–20 positioning system. The ground electrode was placed on the near Fpz location, the EEG signals were acquired with a linked-mastoid reference, and the electrode impedance was kept at less than 5 kΩ.

Signals from all channels were amplified, filtered (0.53~150 Hz), and then digitised with a sampling frequency of 250 Hz. Resting-state EEG data were recorded for 4 min with the eyes closed. As preprocessing steps, the EEG data were high-pass filtered offline above 1 Hz and recomputed to the common average reference. Artifacts were removed with an independent component analysis. Following the removal of artifacts from the EEG signals, source reconstruction was performed using FieldTrip software^43^. Additionally, the boundary element method and a beamforming approach were applied to the source reconstruction.

Since the primary aim of the present study was to investigate the effects of MBT on the DMN, the regions of interest (ROIs) in the DMN were selected prior to the calculation of FC values. Based on previous studies that investigated the DMN using EEG^32–34^, the following 10 ROIs were selected as components of the DMN: left/right middle frontal gyrus (MFG_L/R), left/right middle temporal gyrus (MTG_L/R), posterior cingulate cortex (PCC), anterior cingulate cortex (ACC), left/right hippocampal formation (HF_L/R), and left/right inferior parietal lobule (IPL_L/R). Table 3 presents summary information about the ROIs, including the Montreal Neurological Institute (MNI) coordinates, anatomical regions, corresponding Brodmann areas, and abbreviations.

**Table 3:** List of anatomical regions of interest (MNI coordinates, anatomical regions, abbreviation, Brodmann area (BA).

ROI	MNI coordinates			Anatomical regions	Abbreviation	BA
1	−40	35	35	Left Middle Frontal Gyrus	MFG_L	9
2	35	40	30	Right Middle Frontal Gyrus	MFG_R	9
3	−55	−15	−15	Left Middle Temporal Gyrus	MTG_L	21
4	55	−15	−10	Right Middle Temporal Gyrus	MTG_R	21
5	−5	−50	20	Posterior Cingulate Cortex	PCC	23
6	−5	40	20	Anterior Cingulate Cortex	ACC	32
7	−25	−25	−20	Left Hippocampal Formation	HF_L	28
8	25	−20	−20	Right Hippocampal Formation	HF_R	28
9	−55	−30	35	Left Inferior Parietal Lobule	IPL_L	40
10	55	−30	35	Right Inferior Parietal Lobule	IPL_R	40

Imaginary coherence (iCoh) was employed as a measure of FC because it is regarded as an excellent measure for the study of brain interactions and has minimal volume conduction issues. The iCoh was defined as follows:

1 $${\rm{iCoh}}={\rm{im}}({\rm{Coh}}({f}))={\rm{im}}(\frac{{{S}}_{{xy}}({f})}{{({{S}}_{{xx}}({f}){{S}}_{{yy}}({f}))}^{1/2}})$$

In equation 1, *S* indicates the cross-spectrum and ‘im’ denotes the imaginary part of the coherency (Coh[*f*]). By using the imaginary part of coherency, the iCoh is able to robustly capture phase differences between two time series; detailed equations are available elsewhere. The iCoh values in five frequency bands, delta (1~4 Hz), theta (4~8 Hz), alpha (8~13 Hz), beta1 (13~21 Hz), and beta2 (21~30 Hz) were calculated for each subject. Next, the global FC values between the MBT and healthy control groups were evaluated by summing individual FC matrices and then averaging by group. Global FC values can be regarded as an indicator of DMN strength (i.e., a higher value for global FC indicates stronger connectivity within the DMN).

### Statistical Analysis

#### Psychological measures

Differences in baseline characteristics between the MBT and HC were compared using independent *t*-tests; the descriptive data are presented in Table 2. A repeated-measures analysis of variance (rmANOVA) was conducted to examine between-group differences in changes in psychological state over time. All analyses were performed using the IBM Statistical Package for the Social Sciences (SPSS, ver. 22), and two-sided *p* values < 0.05 were considered to indicate statistical significance.

#### EEG

A rmANOVA with group and time as factors for each frequency band was performed and Students *t*-tests were used for post hoc analyses. Because there were no significant differences between the groups in terms of FC at baseline (i.e., the first EEG recordings; independent *t*-tests, *p* < 0.05), a rmANOVA was used to test the within-subject interaction effects (time × group at each frequency band) to identify the effects of MBT on the FC network.

#### Power analysis

Effect size was estimated from a previous study^44^. Power analysis was conducted using the “G*Power 3” software^45^. With an alpha level of 0.05, 12 cases per group were required to detect a moderate effect size (Cohen’s *d* = 0.54) with adequate power (1-*β* = 0.9).

## Electronic supplementary material

Supplementary Note

## Data Availability

The datasets generated during and/or analysed during the current study are available from the corresponding author on request.
