# Supplementary material for: Effects of an Online Mind–Body Training Program on the Default Mode Network: An EEG Functional Connectivity Study
Source: Sci Rep. 2018 Nov 16;8:16935. doi: 10.1038/s41598-018-34947-x (PMC6240056; doi:10.1038/s41598-018-34947-x)
Supplement: Supplementary file 1 — Supplementary Note [file 41598_2018_34947_MOESM1_ESM.pdf]

Supplementary Note

Effects of an Online Mind–Body Training Program on the Default Mode Network: An EEG Functional Connectivity Study

Dasom Lee[1†], Do-Hyung Kang[1,2†], Na-hyun Ha[3], Chang-young Oh[3], Ulsoon Lee[3], Seung Wan Kang[4,5]*

†These leading authors contributed equally to this work.

[1] Department of Psychiatry, Seoul National University College of Medicine, Seoul, Republic of Korea,

[2] Department of Psychiatry, Seoul National University Hospital, Seoul, Republic of Korea,

[3] Department of Brain-based Emotion Coaching, Global Cyber University, Seoul, Republic of Korea,

[4] College of Nursing, Seoul National University, Seoul National University, Seoul, Republic of Korea,

[5] Data Center for Korean EEG, College of Nursing, Seoul National University, Seoul, Republic of Korea

* Corresponding author

Seung Wan Kang, MD & PhD

College of Nursing, Seoul National University, 103 Daehak-no, Chongno-gu, Seoul, Republic of Korea. 03080. Tel: +82-2-740-8807, +82-2-747-742; Fax: +82-2-745-7422; e-mail: drdemian@snu.ac.kr

Training Program Procedure

The MBT program was performed by the experimental group at home or at the workplace for 8 weeks. Those in the experimental group participated individually in an online MBT program at home or at their workplace for 8 weeks. The program was performed once per day, 5 days per week, with each daily session lasting 10 minutes. Participants were taught the techniques by watching MBT experts in an online video and then followed the MBT protocol for each 10-minute program. Participants in the MBT program were provided with a checklist on which to record their daily practice, which they submitted after the 8-week training. The program was based on mind–body training, a kind of movement-based meditation designed to facilitate relaxation of the mind and release of negative emotions through natural rhythmic movements with a focus on bodily sensations. The program consists of 10 phases: brain relaxation exercise 1, brain relaxation exercise 2, brain rejuvenation exercise, relaxation breathing, chest breathing, meditation with self-watching, energy-focused meditation, brain-refreshing meditation, meditation for balanced brainwaves, and meditation for emotional release.

The first phase, brain relaxation exercise 1, was designed to enhance flexibility and blood circulation by stretching the muscles. It consists of postures and motions that help relax the neck, shoulders, and lower back, which can easily become tight when workers are exposed to conditions associated with burnout.

The second phase, brain relaxation exercise 2, was also designed to enhance flexibility and blood circulation by stretching the muscles. Additionally, this exercise included movements that pulled the tips of the toes toward the body and then pushed them outward, and bringing the hands into the armpits and dropping them down while relaxing the tension in the shoulders.

The third phase, the brain rejuvenation exercise, rejuvenated the relaxed muscles and increased the energy level of the body. It consisted of exercises, such as body tapping, toe-tip tapping, and clapping hands.

In the fourth phase, relaxation breathing was used to deepen and slow the breathing cycle, which becomes short and shallow due to stress. It consisted of taking one breath in six segments and breathing out quickly. Both this breathing exercise and its reverse were repeated three or four times.

In the fifth phase, chest breathing was performed to reduce emotional stress by integrating breathing and consciousness. Focus was on the chest when breathing in and on the abdomen when breathing out. This exercise was repeated four or five times, allowing breathing to become more comfortable. It is easier to focus on and follow this exercise if the right hand is placed on the chest and the left hand is placed on the lower abdomen.

The sixth phase, meditation with self-watching, is performed to relax muscular tension by focusing on the body. Participants focused on each part of the body to increase relaxation as the instructor named it, moving from the top of the head to the tips of the toes. This exercise is repeated two or three times.

The seventh phase, energy-focused meditation, involves feeling the energy of the body and establishing peace of mind. It consists of the motions needed to move the hands 5 cm above the chest, move them apart, and move them closer while feeling the sensation of one’s palms.

The eighth phase, brain-refreshing meditation, is employed to clear the stress-related fogginess of the brain. As participants assume a meditation posture, moving the hands apart and closer, they imagine and say the following: “Pure breath is coming into my head, and my brain is expanding.” They then breathe out with a “whoo” sound and imagine, “Foggy gas is coming out from my brain through my mouth.” It is important that a positive brain state is imagined.

The ninth phase, meditation for balanced brainwaves, is aimed at converting the beta waves associated with stress to alpha waves associated with relaxation and concentration. The first step involves breathing in, holding the breath, and relaxing the neck as the breath is released. The second step involves moving or shaking the head from side to side in a comfortable and relaxing manner. The third step consists of quietly stopping all motion and relaxing the mind.

The tenth phase, meditation for emotional release, was designed to erase and reduce the influence

of images of stressful events or people stored in the brain. First, participants recalled an image of a negative experience and noticed its position and size. They used their imagination to make it as small as a pea and blow it away while saying, “whoo”. This was repeated several times. The experimental subjects repetitively practiced each of the 10 phases four times over the course of 8 weeks.
